# Manganese-Oxide-Modified Porous Carbon from *Ulva prolifera* for Efficient Cd(II) Removal: Adsorption Behavior and Mechanisms

**DOI:** 10.3390/molecules31193509

**Published:** 2026-10-01

**Authors:** Yuhang Zhang, Baoli Wang, Xiujiu Yang

**Affiliations:** Haikou Key Laboratory of Marine Contaminants Monitoring Innovation and Application, Haikou Marine Geological Survey, China Geological Survey, Haikou 571127, Chinabaoli_0916@163.com (B.W.)

**Keywords:** biomass, *Ulva prolifera*, porous carbons, activated carbon, heavy metals, adsorption

## Abstract

Biochar materials exhibit promising prospects in adsorption–separation owing to their large specific surface area and well-developed pore structures. Herein, a series of manganese-oxide-modified porous carbon adsorbents (PEC-MnO_x_) were fabricated via the pyrolysis method, using *Ulva prolifera* as the carbon source and KMnO_4_ as the activator. XRD, SEM, TEM, N_2_ adsorption–desorption, FT-IR and XPS were employed to characterize the morphology, crystal phase, pore structure and surface chemical properties of the as-prepared materials. The results demonstrated that KMnO_4_ activation effectively optimized pore architecture, increased specific surface area, and generated abundant unsaturated –Mn–OH sites and oxygen vacancies. Batch adsorption experiments were carried out to evaluate Cd(II) removal performance. PEC-MnO_x_-6 displayed the optimal adsorption capability with an equilibrium adsorption uptake of 152.4 mg/g at 298.15 K. Kinetic fitting suggested that the pseudo-first-order model better described the adsorption behavior. Intraparticle diffusion analysis confirmed the multi-step mass-transfer nature, and liquid-film diffusion acted as the primary rate-limiting step. Both Langmuir and Freundlich isotherm equations obtained high correlation coefficients, indicating the coexistence of homogeneous active sites and heterogeneous pores/defects on the carbon matrix. Combined with characterization results, Cd(II) sequestration was realized through synergistic effects mainly including surface complexation and ion exchange, with the assistance of electrostatic interaction. This work provides a reference for high-value utilization of *Ulva prolifera* biomass and remediation of Cd(II)-contaminated wastewater.

## 1. Introduction

With the rapid advancement of global industrialization, industries such as mining, smelting, electroplating, chemical processing, and electronics manufacturing have injected strong momentum into economic development while also generating large amounts of industrial wastewater containing heavy-metal ions (such as lead, cadmium, chromium, mercury, and arsenic) [1,2,3,4,5]. These heavy-metal ions are characterized by their non-biodegradability, bioaccumulation, and high toxicity. If discharged directly into water or soil environments without effective treatment, they will accumulate through the food chain, causing severe damage to the structure and function of ecosystems, as well as posing a significant threat to human health [6,7,8,9,10]. For example, lead ions can damage the human nervous system, digestive system, and hematopoietic system, particularly causing irreversible effects on children’s intellectual development [11,12]. Long-term accumulation of cadmium ions can lead to kidney damage and bone disorders, triggering “kodakushima” [13,14]. Chromium ions are recognized as strong carcinogens, which can enter the human body through respiratory, digestive, and skin contact, inducing various cancers [15,16,17]. The treatment of heavy-metal pollution has become one of the critical issues urgently needing resolution in the field of environmental protection, and the development of efficient, economical, and environmentally friendly heavy-metal wastewater treatment technologies holds significant practical importance and urgency [18].

Among various heavy-metal wastewater treatment technologies, the adsorption method has gained widespread application in practical engineering due to its advantages of simple operation, high treatment efficiency, good selectivity, relatively low cost, and ease of resource recovery [19,20]. The core of the adsorption method lies in the selection of adsorbent materials. Traditional adsorbents such as activated carbon (derived from coal, petroleum coke, etc.) [21], zeolites [22], and montmorillonite [23,24,25], while possessing certain adsorption capabilities, face limitations including high raw material costs, high energy consumption during preparation, poor adsorption selectivity, or suboptimal regeneration performance, which restrict their large-scale application in heavy-metal wastewater treatment. Consequently, the development of novel, highly efficient, environmentally friendly, and widely available adsorbent materials has become a focal point in the field of adsorption research.

Biomass-derived activated carbon has unique advantages, such as large specific surface area, well-developed pore structure, tunable surface chemical properties, broad availability, excellent environmental compatibility, and biodegradability or recyclability. These features make it highly promising for heavy-metal ion adsorption [26,27]. At present, a great deal of research has been conducted on the preparation of biomass-derived activated carbon and its adsorption performance for heavy-metal ions. In terms of preparation, the types of biomass raw materials, pretreatment methods, activation processes, and the types of activators significantly influence the pore structure and surface chemical properties of biomass-derived activated carbon. Biomass-derived activated carbon prepared by chemical activation methods (e.g., KOH activation) typically exhibit a larger specific surface area and more abundant microporous structures [28], while physical activation methods (e.g., CO_2_ activation) are more conducive to regulating the ratio of mesopores to macropores [29]. Different activators also introduce various functional groups onto the surface of activated carbon. These functional groups significantly affect the adsorption uptake and selectivity of heavy-metal ions through complexation, ion exchange, and electrostatic adsorption interactions with heavy-metal ions. For example, Wang et al. [30] used walnut shell waste as the raw material and carried out two-step chemical activation with phosphoric acid to successfully synthesize highly efficient nickel-doped porous biochar (PCNi3). The results showed that H_3_PO_4_ activation and nickel doping treatment could effectively improve the microstructure characteristics, thereby enhancing the Cr(VI) adsorption uptake. ZnCl_2_ can be used as a sintering aid and as an activator to construct a hierarchical structure. A ZnCl_2_-modified biochar ZBC-3 was prepared with a specific surface area of 788 m^2^ g^−1^ and a mesopore ratio of 30.92%, and it contains functional groups rich in nitrogen and oxygen elements. Moreover, doping Zn^2+^ into the biochar helps to make it positively charged, thereby facilitating the electrostatic adsorption of Cr(VI) [31].

*Ulva prolifera* is a large green alga with broad salt- and temperature-tolerance. It can attach to intertidal rocks for growth or detach from substrates and drift with seawater. Massive blooms of *Ulva prolifera* block underwater sunlight, deplete dissolved oxygen, cause aquatic organism mortality, and damage coastal ecosystems, while also obstructing watercourses, polluting shorelines and increasing coastal cleaning and management costs. Although several attempts have been reported to convert *Ulva prolifera* into biochar adsorbents, most existing studies merely adopt direct pyrolysis without targeted modification. There remains insufficient understanding regarding how manganese-oxide modification regulates pore architecture and surface active sites of *Ulva prolifera*-derived carbon, and the intrinsic Cd(II) removal mechanism of such composites still lacks systematic clarification. The present work proposes a facile pyrolysis-roasting strategy to fabricate manganese-oxide functionalized porous carbon from waste *Ulva prolifera* biomass, with the following innovations: (1) KMnO_4_ was employed as both activator and modifier to synchronously optimize pore structure and construct abundant unsaturated –Mn–OH active sites and oxygen vacancies on carbon matrix; (2) multi-scale characterizations combined with batch adsorption experiments revealed the structure–activity relationship between material micro-properties and Cd(II) adsorption performance; (3) the multi-step mass-transfer feature and synergistic adsorption mechanism dominated by surface complexation-ion exchange were further deciphered. Scanning electron microscopy (SEM), transmission electron microscopy (TEM), X-ray diffraction (XRD), Brunauer–Emmett–Teller (BET) analysis, X-ray photoelectron spectroscopy (XPS), and Fourier-transform infrared spectroscopy (FT-IR) were adopted to analyze the microstructure, crystal phases, pore features and surface functional groups of as-prepared carbon composites. The influences of pH, adsorbent dosage, initial Cd(II) concentration and contact time on adsorption performance were systematically explored. Combined with adsorption kinetics and isotherm fitting, the underlying heavy-metal adsorption mechanism was elucidated. This research not only provides a feasible technical route for high-value resource utilization of bloomed *Ulva prolifera*, advancing the modification theory of algae-derived porous carbon, but also delivers a low-cost and eco-friendly adsorbent candidate for remediating cadmium-polluted wastewater.

## 2. Experimental

### 2.1. Chemicals

The *Ulva prolifera* was collected from the coastal area of Wenchang, Hainan. Potassium hypermanganate (KMnO_4_, Sigma Aldrich 99.99% purity) used in this experiment is analytically pure without further purification. The standard solutions of Pb(II) and Cd(II) were purchased from Beijing Tanmo Quality Inspection Technology Co., Ltd. (Beijing, China). The ultrapure water was filtered by Milli-Q IQ 7000 (Merck Millipore, Darmstadt, Germany) and used throughout the entire experiment.

### 2.2. Apparatus and Measurements

Scanning electron microscopy (SEM) images were taken on a JSM-7600F (JEOL, Akishima, Tokyo, Japan) microscope and transmission electron microscopy (TEM) was carried out using a JEM-2010F (JEOL, Akishima, Tokyo, Japan) microscope operated at 200 kV. N_2_ adsorption–desorption analysis was measured on a surface area and porosity analyzer (ASAP 2020, Micromeritics, Norcross, GA, USA), with the specific surface area and the pore size distribution deduced by using Brunauer–Emmett–Teller (BET) theory and Barrett–Joyner–Halenda (BJH) analysis, respectively. The total pore volume was obtained from nitrogen adsorption at P/P_0_. Raman spectra of the materials were analyzed by LabRam Micro Raman Spectrometer (Jobin-Yvon HR 800 UV, Longjumeau, France) by using a 514 nm laser excitation source. X-ray photoelectron spectroscopy (XPS) was performed on an AXIS HIS 165 spectrophotometer (Kratos Analytical, Manchester, UK). X-ray diffraction (XRD) experiments were performed on a D/Max-2500 V X-ray diffractometer (Rigaku, Akishima, Tokyo, Japan) with Cu-Kα_1_ radiation. The total amount of heavy-metal ions was determined by ICP-OES (ICAP6300 Radial, Thermo-Fisher Scientific Inc., Waltham, MA, USA).

### 2.3. Preparation of Ulva prolifera-Derived Nanocomposites

**Scheme 1:**
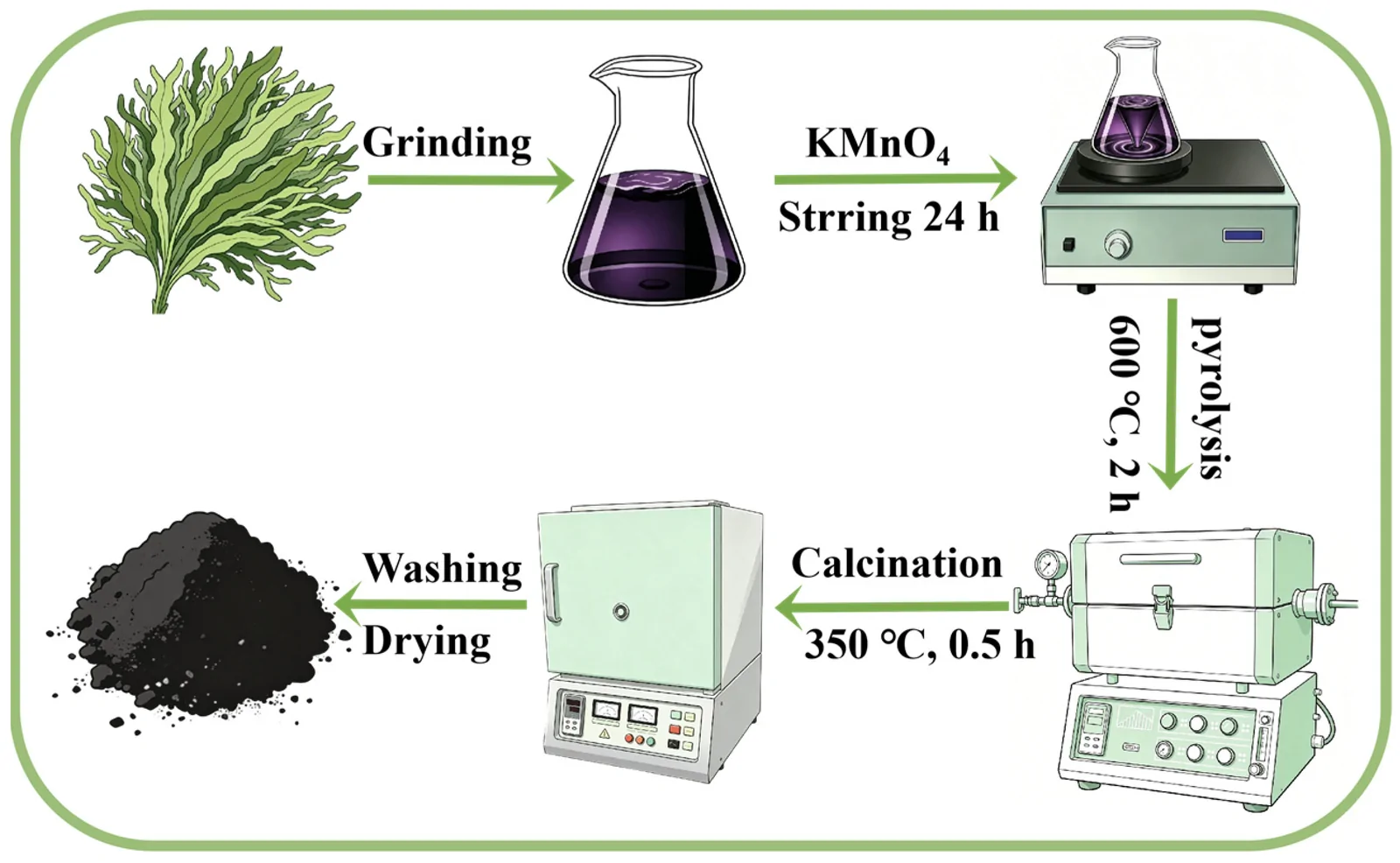
The preparation process of PEC-MnO_x_.

*Ulva prolifera* was washed with water and dried at 90 °C for 24 h, then it was crushed and sieved through an 80-mesh sieve. Next, 5 g of the powder was mixed with 0.2 g, 0.4 g, 0.6 g and 0.8 g KMnO_4_ in the beaker, respectively, and 60 mL water was added with continuous stirring for 12 h. The mixture was then dried at 100 °C for 48 h followed by carbonizing in a tube furnace under an Ar atmosphere at 600 °C with a rate of 5 °C min^−1^ and maintained for 2 h. The carbonized material was subjected to calcination at 350 °C for 30 min, followed by soaking in 1 mol/L NaOH to remove the impurities. Finally, the obtained materials were washed with ultrapure water until neutral and dried at 60 °C overnight to obtain the samples with the names PEC-MnO_x_-2, PEC-MnO_x_-4, PEC-MnO_x_-6, and PEC-MnO_x_-8, respectively (Scheme 1). For comparison, PEC was prepared using the same method, except that no KMnO_4_ was added in the procedure.

### 2.4. Adsorption Experiments

Batch adsorption experiments were carried out to evaluate the adsorption uptake of heavy metals on PEC, PEC-MnO_x_-2, PEC-MnO_x_-4, PEC-MnO_x_-6, and PEC-MnO_x_-8. Each experiment used 40 mg of the material and 50 mL metal solution with a concentration of 200 mg/L. To achieve adsorption equilibrium, the above system was shaken at 150 rpm for 5 h. To explore the adsorption kinetics, 40 mg of different samples were added to a conical flask containing 50 mL of simulated Cd(II) waste solution at a dosage of 0.8 g/L. The initial pH value of the solution was set to 4. At predetermined intervals (0–3000 min), 0.5 mL of the suspensions were collected and then filtered through a 0.45 μm nylon filter membrane. The residual metal concentration in the solutions was quantified by ICP-OES. For adsorption isotherm experiments, the batch adsorption of PEC-MnO_x_ was carried out at Cd(II) concentrations increasing from 40 mg/L to 300 mg/L. The other experimental parameters were the same as those of the kinetics experiments. To investigate the influence of the initial solution pH value on Cd(II) adsorption, PEC-MnO_x_ was placed in simulated solutions of Cd(II) with different initial pH values (pH = 2, 3, 4, 5, 6, 7, 8). The other experimental conditions were the same as those of the kinetics experiments. The adsorption experiments were repeated at least three times. Unless stated otherwise, all adsorption experiments were performed at 298 K.

#### 2.4.1. Removal Percentage and Equilibrium Adsorption Uptake

The removal percentage (*R*) and equilibrium adsorption uptake (*Q_e_*) of metal were calculated by using the following equations:

(1) $$Q_{t}=\frac{(C_{0}-C_{t})\times V}{m}$$

(2) $$Q_{e}=\frac{(C_{0}-C_{e})\times V}{m}$$

(3) $$\mathrm{Removal}\;(\%)=\frac{C_{0}-C_{e}}{C_{0}}\times 100\%=Q_{e}m/C_{0}V$$

where *C*_0_ (mg/L) and *C_t_* (mg/L) represent the Cd(II) concentration before and after adsorption; *C_e_* (mg/L) is the concentration of Cd(II) at equilibrium; *Q_t_* (mg/g) and *Q_e_* (mg/g) denote the adsorption uptake of PEC-MnO_x_ at time and at equilibrium; *m* (mg) stands for the mass of the PEC-MnO_x_; *V* (mL) is the volume of the solution added.

#### 2.4.2. Fitting of Adsorption Kinetics Model

In addition, an intraparticle diffusion model (Equation (4)), pseudo-first-order kinetic model (Equation (5)), pseudo-second-order kinetic model (Equation (6)) and the Elovich model (Equation (7)) were used to fit the kinetic data.

*Q_t_* = *K*_0_*t*^0.5^ + *C* (4)

*Q_t_* = *Q_e_*(1 − *e*^−*K*_1_*t*^) (5)

(6) $$Q_{t}=\frac{Q_{e}^{2}K_{2}t}{1+Q_{e}K_{2}t}$$

(7) $$Q_{t}=\frac{1}{\beta }\ln(1+\alpha \beta t)$$

where *K*_0_ (mg g^−1^ min^1/2^), *K*_1_ (min^−1^) and *K*_2_ (mg g^−1^ min^−1^) are the intraparticle diffusion rate constant, pseudo-first-order rate constant and pseudo-second-order rate constant, respectively; *C* (mg g^−1^) is the constant of intraparticle diffusion; and *α* (mg g^−1^ min^−1^) and *β* (g min^−1^) are the Elovich constants.

#### 2.4.3. Fitting of Adsorption Isotherms

The Langmuir (8) and Freundlich (9) models, as follows, were applied to fit the isothermal data:

(8) $$Q_{e}=\frac{bQ_{m}C_{e}}{1+C_{e}K_{L}}$$

(9) $$Q_{e}=K_{F}C_{e}^{1/n}$$

where *Q_e_* (mg/g) is the adsorption uptake at equilibrium obtained from experimental measurements; *Q_m_* (mg/g) is the adsorption capacity of Cd(II) on the material determined by the Langmuir equation; *K_L_* (L/mg) is the Langmuir constant associated with the affinity of binding sites; *K_F_* (mg/g(L/mg)^1/n^) is the Freundlich constant; and *n* is the heterogeneity factor.

## 3. Results and Discussion

### 3.1. Characterization Analysis

**Figure 1:**
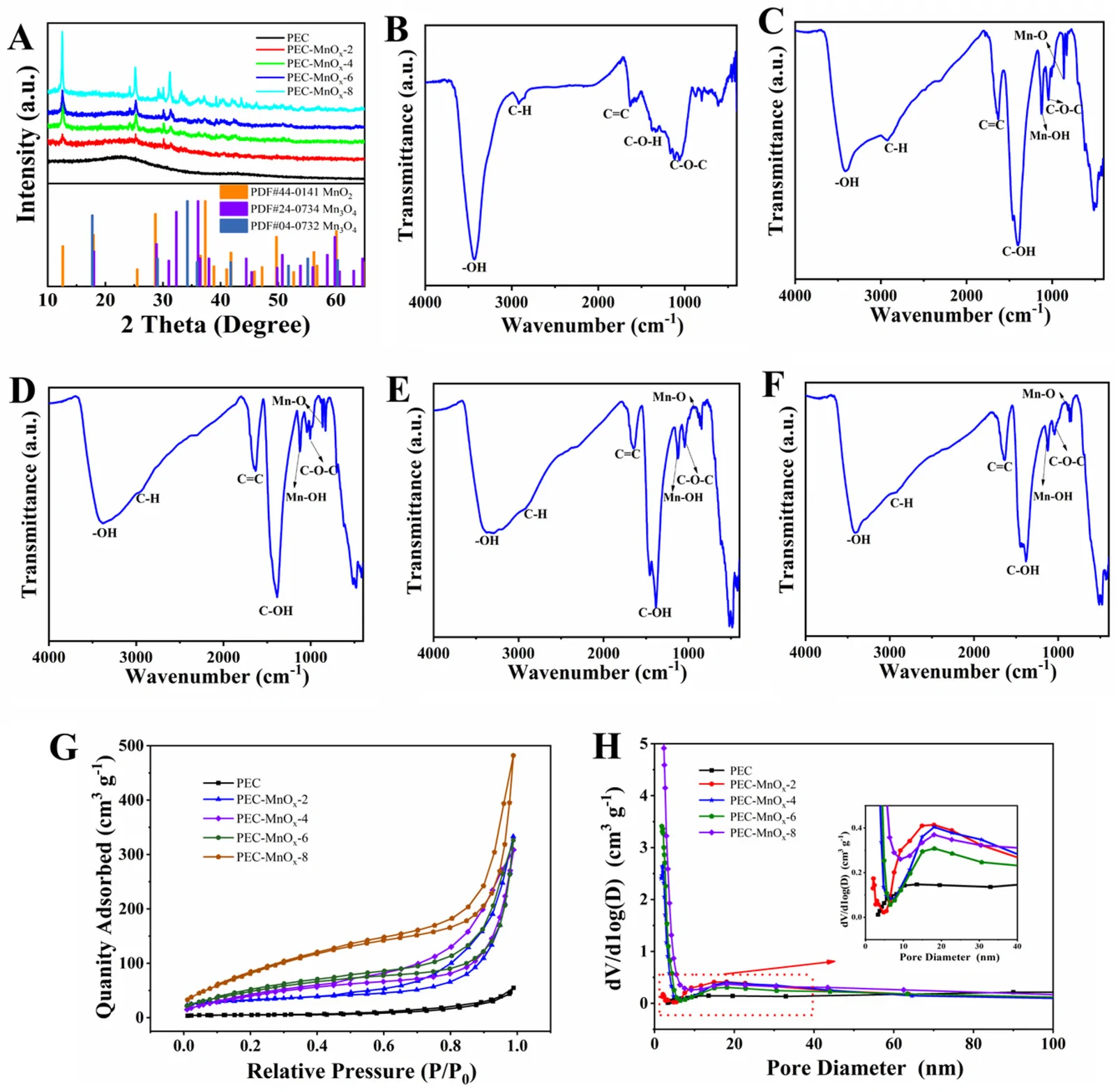
(**A**) XRD patterns; (**B**–**F**) FTIR spectra of PEC, PEC-MnO_x_-2, PEC-MnO_x_-4, PEC-MnO_x_-6 and PEC-MnO_x_-8; (**G**) N_2_ adsorption–desorption isotherms; (**H**) pore size distribution curves.

To characterize the crystal structure of the as-synthesized materials, X-ray diffraction (XRD) measurements were performed, and the corresponding patterns are presented in Figure 1A. As illustrated in the plot, the PEC sample exhibits a broad diffraction peak centered at approximately 24.8°, which is assigned to the (002) crystal plane of graphitic carbon (PDF#41–1487) [32]. Notably, no graphitization catalyst was introduced during the carbonization of PEC. The broadened diffraction peak thus reflects a low graphitization degree, demonstrating that the carbon framework predominantly consists of amorphous carbon. For the PEC-MnO_x_, a distinct set of diffraction speaks at 2θ ≈ 12.5°, 24.8°, 36.7°, and 65.5°, corresponding to the (001), (002), (100), and (110) crystal planes of birnessite-type δ-MnO_2_ (PDF#44–0141). In addition, there are some peaks that belong to Mn_3_O_4_ (PDF#24–0734 and PDF#04–0732). As the addition amount increases, the diffraction peaks gradually become stronger. The average crystallite size of MnO_2_ was calculated by the Scherrer equation:

$$D=\frac{K\lambda }{\beta \cos\theta }$$

where *K* = 0.9, *λ* = 1.5406 Å for Cu Kα radiation, *β* is the full width at half maximum, and *θ* is the Bragg angle. The strongest (001) peak was selected for calculation. The average sizes are about 4.6 nm, 7.3 nm, 10.1 nm and 14.7 nm for PEC-MnO_x_-2, PEC-MnO_x_-4, PEC-MnO_x_-6 and PEC-MnO_x_-8. With increasing Mn loading, the crystallite size of MnO_2_ gradually increases, accompanied by sharper diffraction peaks, which confirms enhanced crystallinity of the manganese oxide phases. Samples obtained at low Mn loading contain very small crystallites with poor crystallinity. Such poorly ordered MnO_x_ structures are susceptible to dissolution or structural collapse under adsorption conditions, leading to leaching and loss of active MnO_x_ components. In contrast, moderately grown crystallites strengthen the structural robustness of MnO_x_, which benefits the cycling stability of the adsorbent.

FTIR spectroscopy was employed to characterize variations in surface functional groups of the as-prepared composites, and the corresponding spectra are displayed in Figure 1B–F. Two prominent absorption bands centered at 3431 cm^−1^ and 1637 cm^−1^ correspond to the stretching vibrations of –OH and C=C bonds, which verify the abundant hydroxyl and carbonyl/carboxyl groups anchored on the material surface, respectively [33]. While the band at 1424 cm^−1^ and 1035 cm^−1^ is characteristic of C–O–H and C–O–C stretching within aromatic carbon rings [34]. The absorption peak at 1117 cm^−1^ is assigned to the stretching vibration of Mn–OH bonds, and the band located at 879 cm^−1^ originates from the bending vibration of hydroxyl groups coordinated to vacant octahedral Mn sites, consistent with the previous literature [35]. Collectively, the FTIR results confirm the successful incorporation of manganese-based species into the carbon matrix, alongside a large quantity of oxygen-containing functional groups distributed over the composite surface.

Figure 1G,H show the N_2_ adsorption–desorption isotherms and matching pore size distribution profiles of the samples. According to the IUPAC classification, all curves show mixed Type II/IV isotherms. A slight increase in the adsorption uptake of the samples was observed at a relative pressure (P/P_0_) below 0.4, which confirms the existence of microporous structures within the materials [36]. Meanwhile, distinct hysteresis loops were detected in the P/P_0_ range of 0.4–1.0 for the samples. This typical adsorption–desorption hysteresis characteristic strongly demonstrates that the prepared materials also possess a well-developed mesoporous structure. The pristine PEC delivers a specific surface area (SSA) of only 71 m^2^/g; in contrast, the PEC-MnO_x_ composites exhibit markedly elevated SSA values after manganese modification. This phenomenon reveals that KMnO_4_ treatment serves as an effective activation strategy to enlarge the SSA of the carbon matrix. Specifically, the measured SSAs of PEC-MnO_x_-2, PEC-MnO_x_-4, PEC-MnO_x_-6 and PEC-MnO_x_-8 are 585, 746, 860 and 806 m^2^/g, respectively. Overall, the SSA rises first and then declines with the gradual increase in MnO_x_ loading content. Such a trend can be explained by pore blockage: excessive manganese oxide nanoparticles will occupy and plug the inner pores of the carbon skeleton, and the inherent surface area provided by MnO_x_ alone fails to offset the loss of pore volume in the carbon substrate [37,38]. For the pristine PEC sample, its pore size distribution is mainly concentrated within the 5.5–7.5 nm range, revealing a hierarchical porous architecture dominated by abundant mesopores alongside a minor fraction of macropores. In comparison, the PEC-MnO_x_ composites display new pore populations centered at approximately 2 nm, which originate from the intrinsic pore channels of immobilized MnO_x_ nanoparticles. Moreover, an obvious pore size distribution peak emerges in the 15–25 nm region, corresponding to interparticle voids formed by the stacking of MnO_x_ species embedded in the carbon matrix. The porous structure parameters of the materials are list in Table 1.

**Figure 2:**
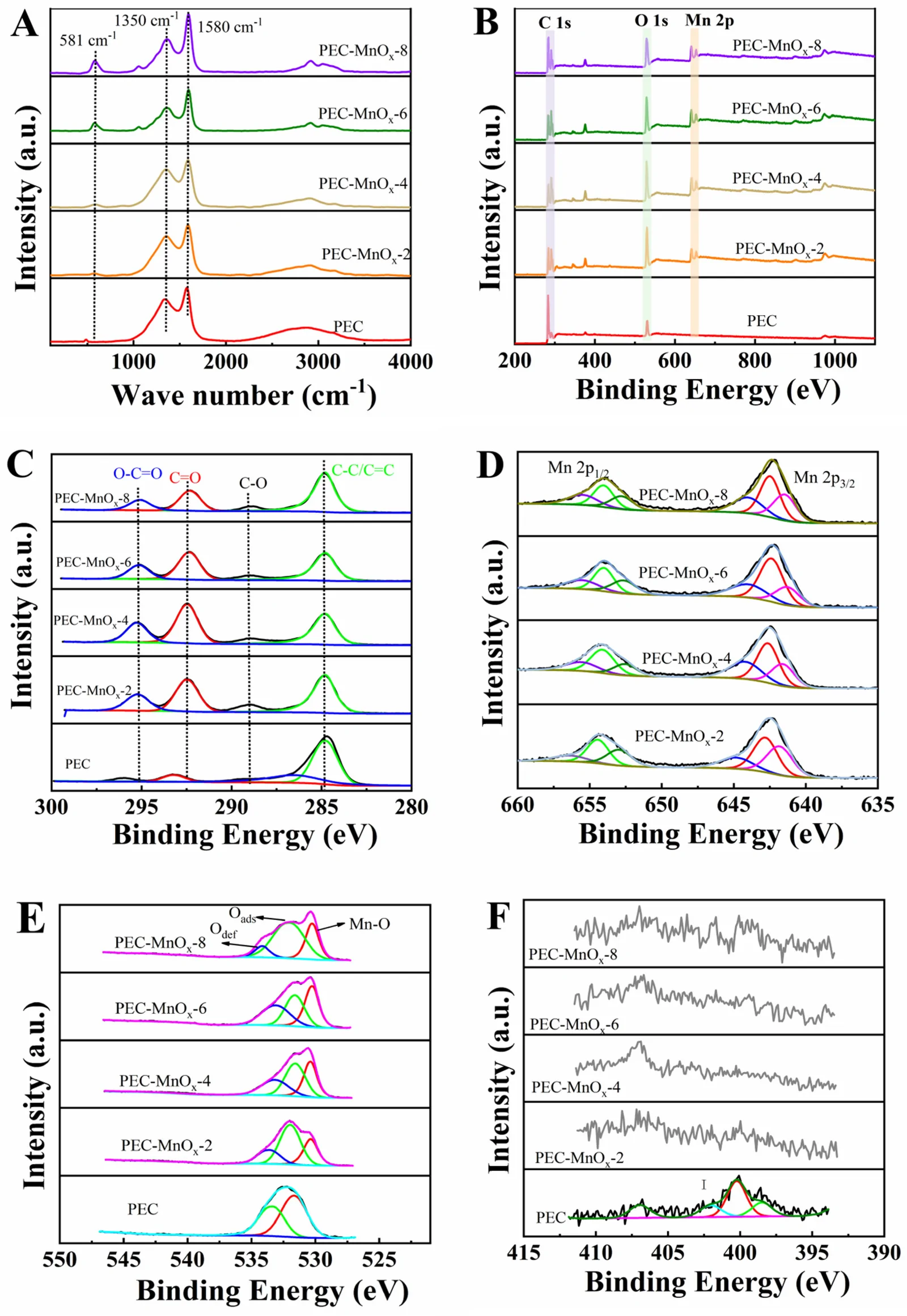
(**A**) Raman and (**B**) XPS spectra of PEC, PEC-MnO_x_-2, PEC-MnO_x_-4, PEC-MnO_x_-6 and PEC-MnO_x_-8. (**C**) C1s, (**D**) Mn 2p, (**E**) O1s and (**F**) N1s spectra.

Raman spectroscopic measurements of the samples were carried out under an excitation wavelength of 514 nm. All spectra (Figure 2A) displayed a D band in the range of 1350 cm^−1^ [39]. This signal arises from edge defects of carbon layers, lattice distortion of sp^2^-hybridized carbon, atomic vacancies, and disorder-induced vibrations originating from impurity doping. The G band, observed at approximately 1580 cm^−1^, corresponds to the in-plane stretching vibration of C-C bonds in sp^2^ carbon, which represents the characteristic vibrational mode of ordered graphitic carbon [40]. The calculated intensity ratio I_D_/I_G_ for PEC, PEC-MnO_x_-2, PEC-MnO_x_-4, PEC-MnO_x_-6 and PEC-MnO_x_-8 gradually decreases. This result suggests that the introduction of manganese oxides elevates the graphitization degree of the carbon support and improves the structural ordering of the carbon framework [41]. Furthermore, no well-defined sharp 2D bands could be identified in the collected spectra. This observation confirms that the samples are predominantly constructed from amorphous carbon and short-range ordered carbon microcrystallites, whereas large-scale stacked graphitic layers are absent within the materials. Additionally, a distinct Raman band centered at ~581 cm^−1^ was observed, which is attributed to the vibration of MnO_x_ [42].

The XPS survey spectra of pristine PEC and PEC-MnO_x_ composites are displayed in Figure 2B. A prominent peak at approximately 285 eV is attributed to the C 1s core-level photoelectron signal of the carbon framework. The band located near 532 eV corresponds to the O 1s signal derived from surface oxygen-containing functional groups on carbon and oxygen species within MnO_x_. Distinct Mn 2p characteristic peaks (Mn 2p_3/2_ and Mn 2p_1/2_ spin–orbit doublet) centered at 638–645 eV and 650–658 eV can be clearly identified for all PEC-MnO_x_ samples [43], whereas no detectable Mn signal appears for pure PEC. This result confirms that MnO_x_ has been successfully immobilized on the PEC carbon substrate. As displayed in Figure 2C, the C 1s spectra were deconvoluted into four sub-peaks assigned to C–C/C=C (~285.0 eV), C–O (~289.5 eV), C=O (~292.0 eV) and O–C=O (~295.0 eV). Compared with pristine PEC, the C–C/C=C peak of PEC-MnO_x_ samples shifts toward lower binding energy. This shift originates from the constructed C–O–Mn heterointerfaces between PEC and MnO_x_, where electrons transfer from MnO_x_ to the carbon framework and increase the electron density of carbon atoms. Meanwhile, the relative contents of oxygen-containing groups (C–O, O–C=O) decrease after modification, demonstrating that surface oxygen functional groups coordinate with Mn species. The increased proportion of sp^2^-hybridized C–C/C=C reveals that KMnO_4_ activation enhances the graphitization degree of the carbon skeleton. The Mn 2p XPS spectra display two characteristic peaks assigned to Mn 2p_3/2_ (638–645 eV) and Mn 2p_1/2_ (650–658 eV) with a spin–orbit splitting energy of ~11.5 eV. The deconvoluted Mn 2p_3/2_ peaks correspond to Mn^2+^ (~641.0 eV), Mn^3+^ (~642.2 eV) and Mn^4+^ (~643.6 eV). The slight positive shift of Mn 2p peaks, combined with the negative shift of C 1s peaks, verifies interfacial electron transfer from MnO_x_ to PEC via constructed C–O–Mn bonds. In addition, the relative proportions of Mn^2+^ and Mn^3+^ increase after pyrolysis activation, indicating partial reduction of Mn^4+^ by the carbon matrix. The mixed-valent manganese oxides generate abundant oxygen vacancies and unsaturated –Mn–OH sites, which serve as active sites for Cd(II) capture via surface complexation and ion exchange. The O 1s spectra were deconvoluted into three peaks, corresponding to lattice oxygen (O_lat_) (~530.5 eV, Mn–O bond), defective/hydroxyl oxygen (O_def_/O_OH_) (~531.6 eV), and adsorbed oxygen (O_ads_) (~532.8 eV), respectively [44]. Only O_OH_ and O_ads_ signals can be detected for PEC, while an obvious lattice oxygen peak emerges after MnO_x_ modification, verifying the successful loading of manganese oxides. Moreover, the relative proportion of O_def_/O_OH_ increases greatly in composites, indicating abundant unsaturated –Mn–OH sites and oxygen vacancies are generated on the surface. These oxygen-containing active sites serve as anchoring centers for Cd(II) via surface complexation and ion exchange. Combined with C 1s and Mn 2p results, the constructed C–O–Mn heterointerfaces induce interfacial electron transfer and modulate the distribution of surface oxygen species. The enriched hydroxyl groups and oxygen vacancies synergistically endow PEC-MnO_x_ composites with outstanding adsorption performance toward Cd(II). The N 1s spectra reveal obvious nitrogen signals for pristine PEC within 395–410 eV, which can be deconvoluted into pyridinic N, pyrrolic N and graphitic N. In contrast, all PEC-MnO_x_ composites exhibit nearly vanished N 1s characteristic peaks. This observation can be explained by two reasons. First, in situ formed MnO_x_ preferentially anchors on nitrogen-containing defect sites of PEC and covers surface N groups, resulting in nitrogen intensity decreasing sharply. Second, permanganate and Mn species exert catalytic oxidation effects, promoting the thermal decomposition of partial unstable edge N, which escapes in the form of nitrogen-containing gas. Although the detectable surface nitrogen declines, the interfacial interaction between intrinsic nitrogen sites and manganese oxides regulates electronic properties, cooperating with Mn–OH and carbon oxygen-containing groups to provide synergistic active sites for Cd(II) capture. Table S1 lists the atomic percentages (at%) of C, Mn, N and O in pristine PEC and PEC-MnO_x_ composites with different MnO_x_ loadings. For the bare PEC sample, no Mn signal was detected, and the atomic fraction of C reached 87.31 at%, accompanied by minor N (2.41 at%) and O (10.28 at%) elements. After MnO_x_ modification, Mn was successfully introduced into the carbon substrate. With the increase in MnO_x_ loading, the Mn atomic percentage gradually rose from 2.91 at% (PEC-MnO_x_-2) to 6.93 at% (PEC-MnO_x_-8). Meanwhile, the C atomic percentage continuously decreased from 66.46 at% to 58.91 at%. The O atomic percentage showed an increasing trend from 30.52 at% to 34.07 at% with higher MnO_x_ loading, which originates from the oxygen in manganese oxides.

**Figure 3:**
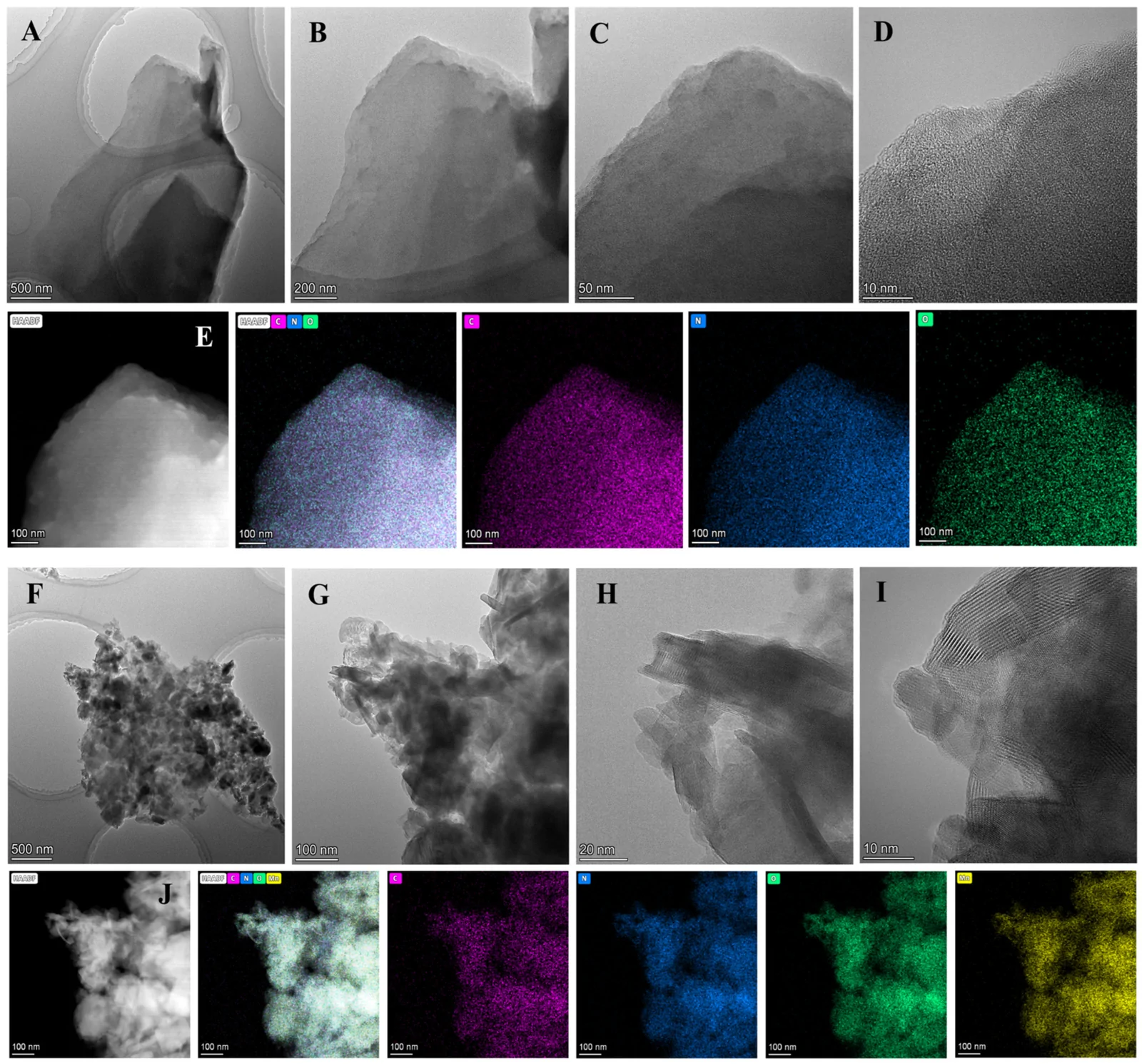
(**A**–**C**) TEM and (**D**) HRTEM images; (**E**) EDS mappings of PEC; (**F**–**H**) TEM and (**I**) HRTEM images; (**J**) EDS mappings of PEC-MnO_x_-6.

TEM was utilized to systematically investigate the microscopic morphological and structural characteristics of the synthesized materials, and the corresponding microscopic images are presented in Figure 3. Prior to MnO_x_ modification, pure PEC exhibits an extended and ultrathin nanosheet structure, as observed from TEM results (Figure 3A–C). High-resolution TEM (HRTEM) images display indistinct lattice fringes for the PEC substrate, which is attributed to its low graphitization degree, confirming that amorphous carbon dominates the carbon framework of PEC. This structural observation is highly consistent with the analytical results of XRD tests. EDS elemental mapping results further verify that PEC is mainly composed of C, N, and O elements, which are homogeneously distributed throughout the entire material architecture. Specifically, the N element is derived from the inherent components of the raw biomass precursor, while the O element originates from the surface oxidation modification of the PEC carbon matrix.

After the successful immobilization of MnO_x_ species on the PEC substrate, the obtained PEC-MnO_x_-6 composite presents distinct morphological differences (Figure 3E–H). As illustrated in Figure 3H, numerous uniform short nanorods with diameters ranging from 10 to 20 nm are firmly anchored onto the PEC carbon skeleton. HRTEM characterization clearly identifies well-resolved lattice fringes corresponding to MnO_2_ and Mn_3_O_4_ crystals, alongside typical graphitic carbon lattice fringes. This phenomenon demonstrates that the introduction of manganese oxide effectively improves the graphitization degree of the PEC carbon matrix. In addition, EDS elemental analysis corroborates the elemental composition of PEC-MnO_x_-6, confirming the coexistence and uniform distribution of C, Mn, O, and N elements in the composite material.

### 3.2. Results of Adsorption Experiments

#### 3.2.1. Effect of Initial Concentration

**Figure 4:**
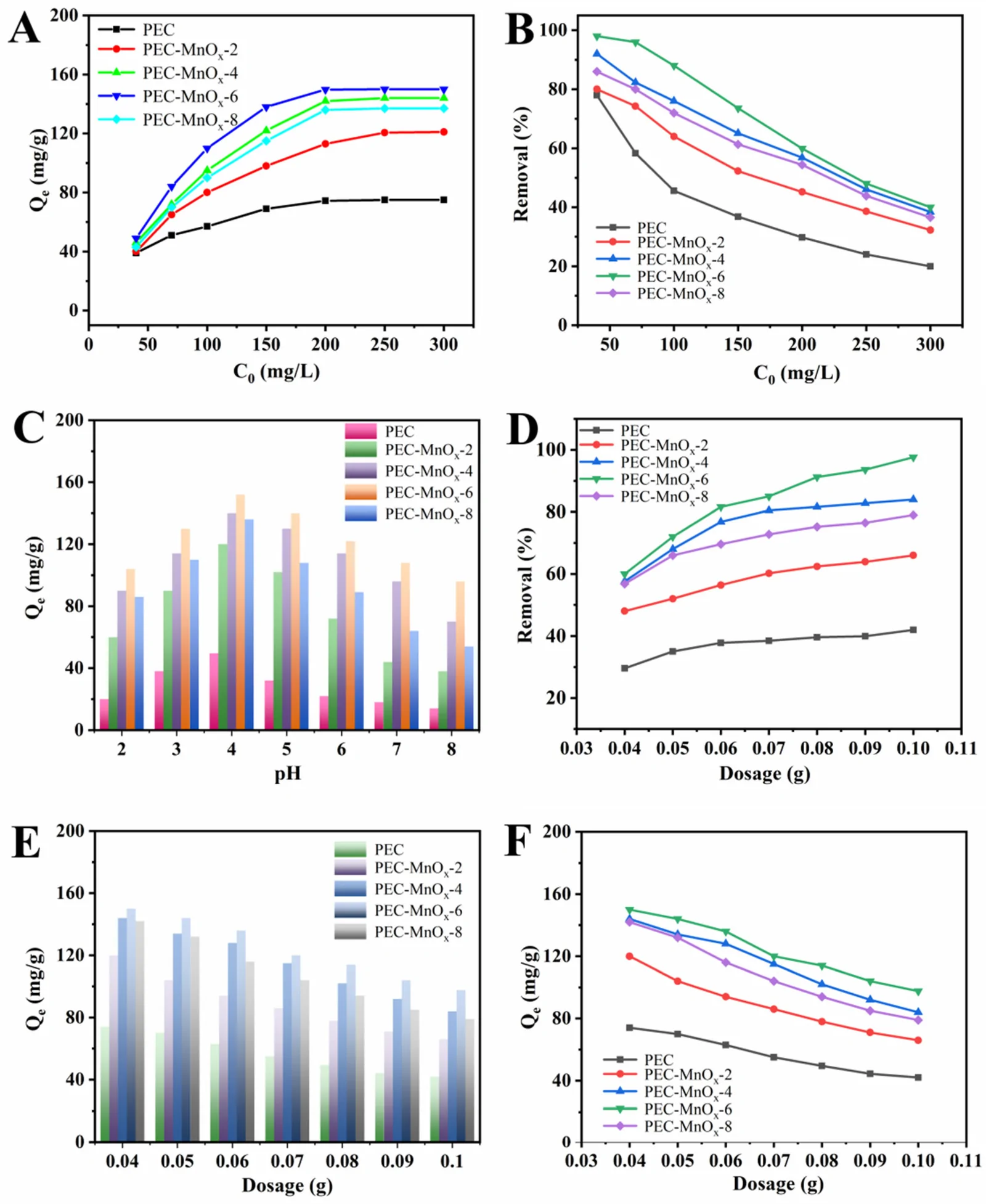
Effect of initial concentration on the Q_e_ (**A**) and removal (**B**) of PEC, PEC-MnO_x_-2, PEC-MnO_x_-4, PEC-MnO_x_-6, and PEC-MnO_x_-8 for Cd(II) adsorption; (**C**) effect of pH on the Q_e_ for Cd(II) adsorption; (**D**) the value of removal for Cd(II) at different dosages of adsorbent; (**E**) the histogram graph; (**F**) line graph of Q_e_ versus different adsorbent dosages.

The equilibrium adsorption uptake of the prepared adsorbents toward Cd(II) displayed a distinct positive correlation with the initial ion concentration of the solution, as clearly illustrated in Figure 4A. Fundamentally, this concentration-dependent adsorption behavior is governed by the mass-transfer driving force of the adsorption system. At low initial Cd(II) concentrations, the concentration gradient between the aqueous solution and the adsorbent surface is relatively small, which limits the diffusion and migration of cadmium ions toward the adsorption interface, resulting in a low saturated adsorption uptake. With the gradual elevation of the initial Cd(II) concentration, a significantly enhanced concentration differential is formed between the liquid phase and the solid adsorbent surface, which provides a powerful thermodynamic driving force to overcome the interfacial mass-transfer resistance and facilitates the effective diffusion and capture of Cd(II) by the adsorbent active sites. When the Cd(II) concentration was increased from 40 mg/L to 300 mg/L at a fixed adsorbent dosage of 0.8 g/L, indicating that there was a significant increase in the adsorption uptake with the increase in concentration. Quantitative experimental data demonstrated that the equilibrium adsorption uptake of PEC-MnO_x_-6 varied significantly with the change in initial Cd(II) concentration, rising steadily from 50.8 mg/g to 152.4 mg/g within the tested concentration range. It consistently delivered the optimal Cd(II) adsorption effect among the five re-synthesized adsorbent samples, further verifying that the reasonable manganese oxide modification strategy can effectively optimize the surface structure and adsorption activity of the carbon matrix, and endow the composite material with excellent heavy-metal ion removal performance. The increased Mn and O content in PEC-MnO_x_-6 confirms the successful immobilization of MnO_x_ nanoparticles on the PEC surface. The enriched oxygen-containing and Mn–OH sites provide abundant binding sites for Cd(II) adsorption, which explains the improved adsorption uptake. Nevertheless, further increasing Mn loading (PEC-MnO_x_-8) does not continuously improve the Cd(II) adsorption performance, even though higher Mn atomic percentage is observed. Excessive MnO_x_ nanoparticles tend to aggregate and block the pore channels of the carbon matrix. Thus, partial Mn–OH active sites are buried inside the aggregated MnO_x_ phases and cannot be accessible to Cd(II) ions in solution. In addition, pore-blocking reduces the available specific surface area, weakening mass-transfer efficiency. Therefore, an optimal MnO_x_ loading exists (PEC-MnO_x_-6), beyond which the adsorption performance declines.

Notably, the initial concentration of 200 mg/L serves as the critical saturation threshold for all prepared adsorbents in this study. When the Cd(II) initial concentration was increased to 200 mg/L, the active sites of each adsorbent were basically fully saturated and the equilibrium adsorption uptake stopped. This might be because the number of residual available adsorption sites decreases sharply, and the mass-transfer driving force generated by the solution concentration gradient can no longer promote further ion adsorption, eventually leading the entire adsorption system to reach a dynamic equilibrium state [45]. This leads to a decrease in the removal of Cd(II) from 98.1% to 59.9% for PEC-MnO_x_-6 (Figure 4B). It is worth noting that the Cd(II) removal efficiency of PEC declines markedly even at low initial concentrations. In comparison, PEC-MnO_x_-6 exhibits only minimal performance attenuation. This observation confirms that appropriate MnO_x_ loading effectively improves the Cd(II) removal performance of PEC-MnO_x_-6. Accordingly, an initial Cd(II) concentration of 200 mg/L was adopted for subsequent experiments.

#### 3.2.2. Effect of Solution pH

The pH of an aqueous solution acts as a vital environmental parameter. It determines the existing species of Cd(II) in water and controls the surface charge characteristics of adsorbents, which fundamentally mediates interfacial interactions during adsorption. The influence of pH (2–8) on Cd(II) adsorption was explored at 25 °C, with an initial Cd(II) concentration of 200 mg/L, adsorbent dosage of 0.8 g/L and solution volume of 50 mL. Cd(II) primarily presents in soluble oxygenated ionic forms in aqueous media under conventional experimental conditions. As illustrated in Figure 4C, the Q_e_ of all five adsorbents peaks at pH = 4. A further rise in pH leads to a decline in Q_e_. Consistently, PEC-MnO_x_-6 delivers the most favorable adsorption uptake among these materials. At pH values below 4, the surface functional groups of PEC-MnO_x_-6 are extensively protonated, enabling the adsorbent to capture target anionic species via electrostatic adsorption. Additionally, the high concentration of H^+^ in strongly acidic aqueous systems facilitates the redox reaction between Mn-based active components and Cd(II) ions. Nevertheless, surface oxidation occurs under elevated pH conditions (pH > 4), which imposes negative impacts on Cd(II) adsorption. Correspondingly, the Q_e_ of PEC-MnO_x_-6 declined from 152.4 mg/g to 96.3 mg/g. Meanwhile, severely acidic conditions (pH ≤ 3) significantly inhibit the Cd(II) adsorption performance of PEC-MnO_x_-6. It can be attributed to the high concentration of chloride ions in strongly acidic media, which competitively occupy the surface active sites of the adsorbent and ultimately weaken the Cd(II) removal effect, which is consistent with the previous literature reports [46].

#### 3.2.3. Effect of Adsorbent Dosage

Adsorbent dosage represents a critical factor governing the quantity of accessible active sites and greatly influences heavy-metal removal efficiency. To clarify how adsorbent dosage regulates Cd(II) elimination, batch experiments were conducted within an adsorbent dosage range of 0.04–0.1 g. Each trial employed 50 mL Cd(II) solution with an initial concentration of 200 mg/L, and the adsorption reaction proceeded for 300 min. Experimental results revealed that pristine biochar exhibited remarkably inferior Cd(II) removal performance compared with MnO_x_-modified composites, verifying the synergistic promotion of MnO_x_ toward Cd(II) adsorption. As illustrated in Figure 4D–F, for PEC-MnO_x_-6, increasing adsorbent dosage leads to a continuous rise in Cd(II) removal efficiency, which originates from the growing number of available adsorption active sites. The removal efficiency gradually levels off and approaches a plateau at higher dosage. In contrast, the equilibrium Q_e_ decreases monotonously with increasing adsorbent loading (Figure 4E,F). This opposite trend is a typical observation in batch adsorption tests: at higher dosages, abundant surplus active sites cannot be fully occupied by limited Cd(II) ions in solution. In addition, particle agglomeration may reduce the effective specific surface area and cause overlapping of adsorption sites. Balancing high removal performance and efficient utilization of adsorption sites, 0.8 g/L was selected as the optimal adsorbent dosage [47]. Therefore, the optimized adsorption conditions were confirmed as an adsorbent dosage of 0.8 g/L, a solution pH of 4, and an initial Cd(II) concentration of 200 mg/L. Under such optimal conditions, the adsorbent attained an adsorption uptake of 152.4 mg/g within 60 min. These optimized experimental parameters were adopted for subsequent exploration of adsorption kinetics and thermodynamics, as elaborated in the following sections.

#### 3.2.4. Effect of Adsorption Time and Kinetics

**Figure 5:**
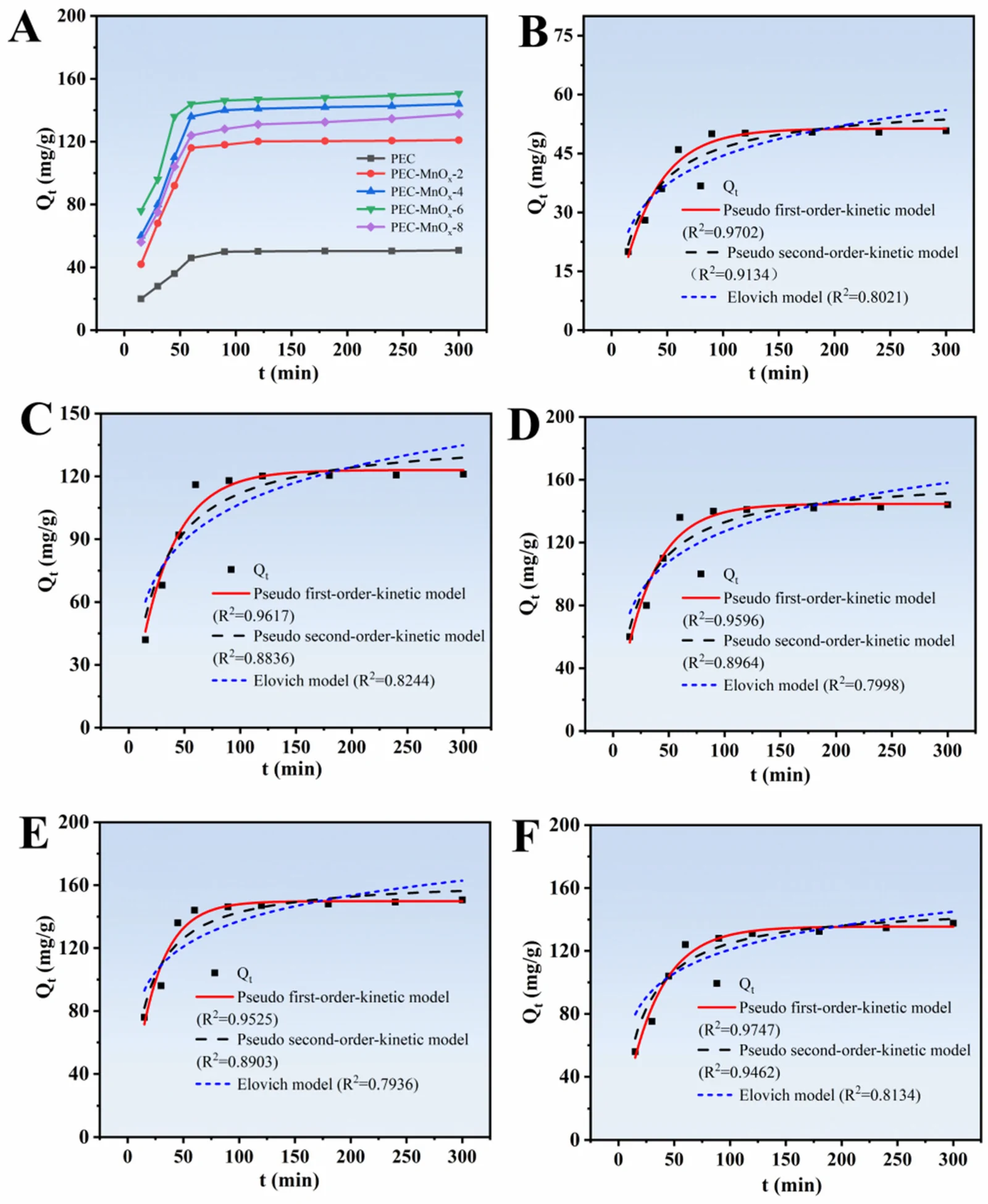
(**A**) Effect of time on the Q_t_ and kinetics models of (**B**) PEC, (**C**) PEC-MnO_x_-2, (**D**) PEC-MnO_x_-4, (**E**) PEC-MnO_x_-6, and (**F**) PEC-MnO_x_-8 for Cd(II) adsorption at 298 K.

Figure 5 quantitatively depicts how varying solid–liquid contact duration modulates the Cd(II) removal performance of the as-synthesized PEC-MnO_x_ adsorbent system, systematically revealing the dynamic adsorption process of Cd(II)onto the material surface and internal pores. From a holistic kinetic perspective, the material displays a two-stage distinct adsorption trend throughout the reaction period (Figure 5A). In the initial rapid adsorption stage spanning the first 60 min of contact, the Cd(II) Qt undergoes a sharp, dramatic surge. This rapid uptake originates from the abundant unoccupied active adsorption sites distributed on the exterior surface of the adsorbent, which are readily accessible to free Cd(II) ions in the aqueous solution without internal diffusion barriers [48]. As the contact time extends to 60 min, the absorption gradually reaches equilibrium, signifying that the majority of available surface binding sites have already captured Cd(II) within a short time frame. Following this fast uptake phase, the growth rate of Cd(II) Q_t_ markedly decelerates and enters a slow adsorption plateau stage. The root cause of this slowed adsorption lies in the progressive saturation of surface functional groups and MnO_x_-based binding sites; with most exterior adsorption positions fully occupied, residual Cd(II) ions can only migrate into the inner pore channels of the carbon substrate through intraparticle diffusion, a process with far slower mass-transfer velocity. The adsorption system ultimately attains complete thermodynamic and kinetic equilibrium at a contact time of 300 min. This comparative advantage powerfully corroborates the outstanding kinetic advantages of the fabricated composite material, including fast ion capture rate, low mass-transfer resistance, and rapid saturation attainment. The time-dependent adsorption experimental results verify that the optimized composite PEC-MnO_x_-6 possesses superior rapid adsorption capability, capable of realizing high-efficiency elimination of aqueous Cd(II). Pristine PEC substrate delivers a relatively inferior maximum Cd(II) adsorption uptake. This deficiency can be reasonably attributed to two intrinsic structural drawbacks of bare PEC, on the one hand, the quantity of surface active functional sites capable of chelating or electrostatically attracting Cd(II) is inherently scarce. On the other hand, the PEC matrix lacks loaded manganese oxide nanoparticles, which serve as the core high-affinity adsorption centers for heavy-metal cations. When tuning the MnO_x_ loading amount on the PEC, the material’s Cd(II) adsorption uptake presents a clear volcano-type variation trend. The capacity ascends continuously at low loading ratios, peaks at an optimal loading amount, and subsequently declines gradually with excessive MnO_x_. This non-monotonic performance evolution can be rationalized by the dual competing effects brought by manganese oxide loading. At moderate loadings, uniformly dispersed MnO_x_ nanoparticles introduce abundant additional active adsorption sites and generate strong synergistic interactions between the carbon carrier and metal oxide, jointly boosting Cd(II) sequestration capacity. Nevertheless, once the MnO_x_ loading exceeds the critical optimal threshold, excessive MnO_x_ particles tend to aggregate and fully cloak the porous framework of the PEC carbon matrix. Such severe pore blockage obstructs the transport channel for aqueous Cd(II) to penetrate into the internal adsorption sites, simultaneously diminishing the interfacial contact area between PEC and MnO_x_, which weakens their synergistic adsorption effect and ultimately triggers a noticeable drop in the overall Cd(II) removal capacity of the composite adsorbent [49,50].

To deeply explore the kinetic behavior of the adsorption process, the pseudo-first-order kinetic model (PFO), pseudo-second-order kinetic model (PSO) and Elovich model were adopted to fit the adsorption kinetic data (Figure 5B–F). The corresponding fitting results show that the correlation coefficient (R^2^) values of the PFO model were 0.9702, 0.9617, 0.9596, 0.9525 and 0.9747 for PEC, PEC-MnO_x_-2, PEC-MnO_x_-4, PEC-MnO_x_-6, and PEC-MnO_x_-8, respectively. Meanwhile, the Elovich model yielded R^2^ values of 0.8021, 0.8244, 0.7998, 0.7936 and 0.8134. Apparently, the PFO model achieved higher R^2^ values than those of the PSO and Elovich models. In addition to higher correlation coefficients, the PFO also presented lower chi-square (χ^2^) statistical errors, further verifying its better fitting performance. These results suggest that the PFO model is more suitable for describing the adsorption kinetic behavior, and it indicates liquid-film diffusion acts as the primary rate-limiting step.

Furthermore, the Weber–Morris intraparticle diffusion model was adopted to investigate the diffusion behavior of Cd(II) within the adsorbent. As illustrated in Figure S1, the kinetic curves display two linear regions. The first linear segment is assigned to boundary layer diffusion, while the second one originates from intraparticle diffusion. The third region represents the adsorption equilibrium plateau at saturation. Notably, the plots of Q_t_ versus t^1/2^ yield non-zero intercepts, indicating that intraparticle diffusion is not the only rate-limiting step. Instead, liquid-film diffusion and internal mass transfer collectively control the overall adsorption kinetics. For comparison, the pure PEC sample only exhibits one linear segment in the Weber–Morris plot. The initial steep linear region corresponds to rapid Cd(II) uptake, where Cd(II) ions cross the liquid film and occupy external surface active sites. Subsequently, the curve quickly plateaus. No distinguishable intraparticle diffusion stage can be observed, indicating that adsorption rapidly reaches dynamic equilibrium once the external active sites are occupied.

#### 3.2.5. Effect of Temperature and Adsorption Isotherms

**Figure 6:**
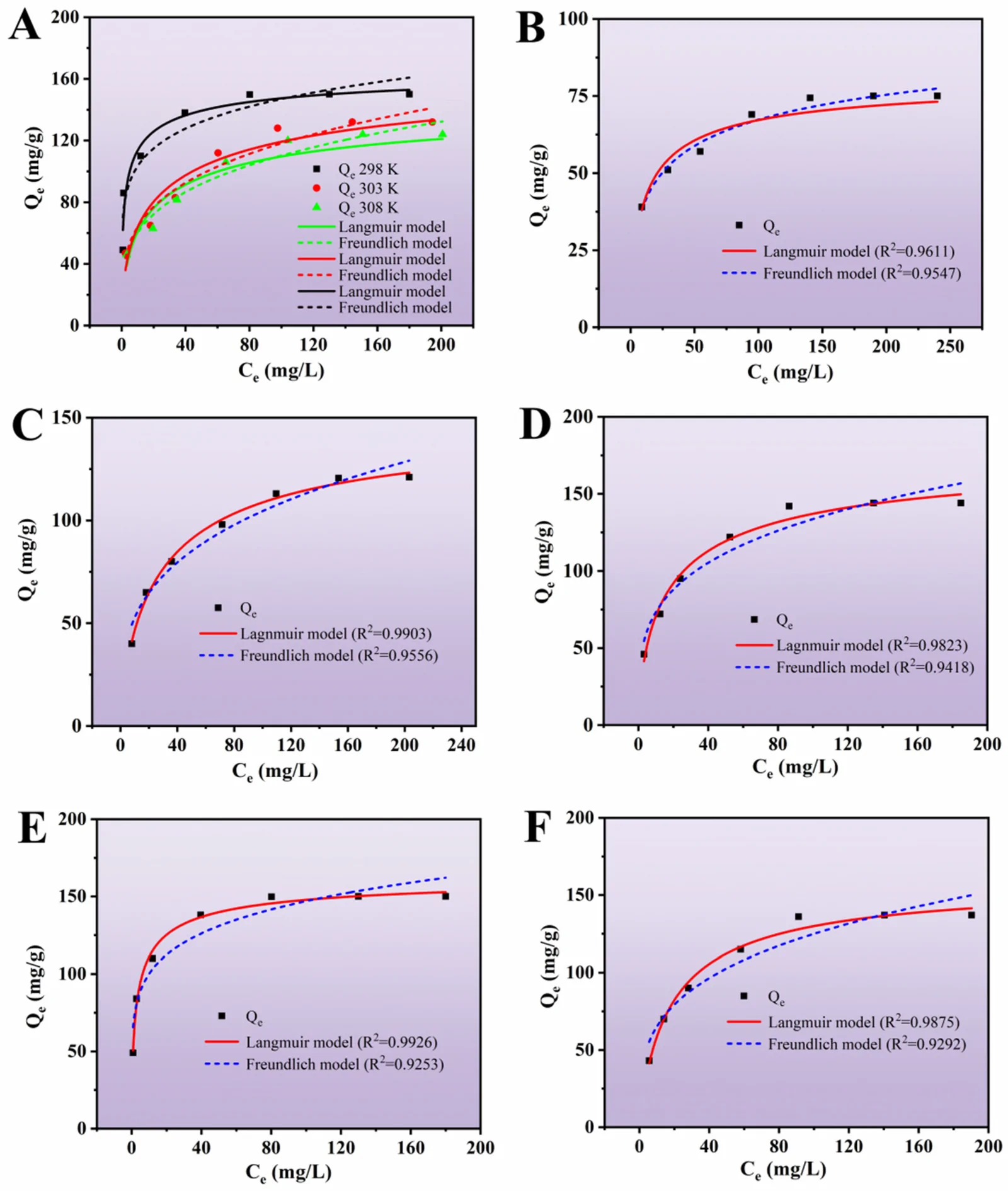
(**A**) Adsorption isotherms for Cd(II) adsorption by PEC-MnO_x_-6 at different temperatures; adsorption isotherms for Cd(II) adsorption by (**B**) PEC, (**C**) PEC-MnO_x_-2, (**D**) PEC-MnO_x_-4, (**E**) PEC-MnO_x_-6 and (**F**) PEC-MnO_x_-8 at 298 K.

Since PEC-MnO_x_-6 possessed the optimal adsorption performance among all prepared adsorbents, it was selected for investigating the temperature effect on Cd(II) adsorption. Figure 6 illustrates the influence of reaction temperature (25–35 °C) on equilibrium adsorption performance toward aqueous Cd(II). Experimental results revealed a negative temperature-dependent trend for PEC-MnO_x_-6: the equilibrium adsorption uptake for Cd(II) decreased from 152.4 mg/g at 298 K to 128.9 mg/g at 308 K. Nevertheless, the reduction in adsorption uptake was relatively limited within the tested temperature range. No sharp drop in Cd(II) adsorption uptake was observed upon temperature elevation, indicating that temperature was not a dominant factor controlling the adsorption behavior of the as-prepared composites under the experimental conditions. The decrease in uptake capacity at higher temperatures implies that the overall adsorption process is exothermic. It may result from physical intermolecular interactions, as well as the strengthened desorption tendency of pre-adsorbed Cd(II) ions.

As shown in Figure 6C–F, the Langmuir isotherm equations yields a slight higher R^2^ than the Freundlich isotherm equations for all PEC-MnO_x_ samples, indicating that the experimental data are better described by the Langmuir isotherm equations. Nevertheless, the Freundlich isotherm equations also yield relatively high R^2^ values, demonstrating that the surfaces of PEC-MnO_x_ composites are not perfectly homogeneous. This reflects the inherent heterogeneous nature of the adsorbent, which arises from its complex pore architecture and diverse surface functional groups. Therefore, Cd(II) adsorption onto PEC-MnO_x_ composites is dominated by monolayer adsorption on homogeneous active sites, accompanied by adsorption onto heterogeneous-surface sites. For pristine PEC, the comparable R^2^ values of the Langmuir and Freundlich equations suggest potential multilayer-dominated adsorption characteristics. Cd(II) adsorption on bare PEC mainly relies on van der Waals-driven physical interactions, although minor chemical contributions originating from surface oxygen-containing groups cannot be ignored. After MnO_x_ loading, abundant and uniformly distributed Mn–OH active sites are introduced onto the carbon substrate, and the adsorption mechanism shifts toward coupled physico-chemical adsorption. Since chemisorption takes place over finite accessible active sites, further molecular stacking is restricted once these sites become saturated. Meanwhile, the deposited MnO_x_ may partially block pores and cover intrinsic carbon-based adsorption sites, thus weakening the contribution of multilayer physical adsorption.

### 3.3. Proposed Mechanism of Adsorption

The efficient removal of aqueous Cd(II) by PEC-MnO_x_-6 composite is realized through the synergistic combination of chemical-dominated adsorption behaviors, auxiliary electrostatic interaction and weak physical capture. Abundant unsaturated Mn–OH sites originating from mixed-valent manganese oxides (Mn^2+^, Mn^3+^ and Mn^4+^), together with inherent oxygen-containing functional groups (–COOH, C–OH and C=O) on the *Ulva prolifera*-derived carbon skeleton, serve the major reactive centers for Cd(II) sequestration. Under appropriate solution pH conditions, these surface –OH undergo deprotonation and generate negatively charged surface sites, which can form stable inner-sphere surface complexes with positively charged Cd(II) ions via surface complexation. Besides surface complexation, the ion-exchange reaction constitutes another critical chemical adsorption pathway. The exchangeable protons bound to Mn–OH groups on manganese oxide surfaces can be replaced by Cd(II) cations from the aqueous solution, thereby immobilizing heavy-metal ions onto the adsorbent surface.

Electrostatic attraction serves as an auxiliary driving force for Cd(II) enrichment. At optimal pH 4, abundant deprotonated oxygen-containing sites endow PEC-MnO_x_-6 with negative surface charge, which facilitates the attraction and pre-concentration of cationic Cd(II) near the adsorbent-water interface. Nevertheless, strong competitive adsorption from excessive H^+^ under highly acidic conditions will occupy available surface sites and suppress Cd(II) adsorption uptake, which is consistent with the pH-dependent adsorption experimental results. Weak physical adsorption originating from van der Waals interactions of carbon matrix offers only a negligible contribution to the total Cd(II) uptake.

Kinetic and intraparticle diffusion analyses reveal the mass-transfer characteristics during the whole adsorption process. The adsorption proceeds sequentially through liquid-film diffusion, intraparticle diffusion and a final adsorption–desorption equilibrium stage. The superior fitting performance of pseudo-first-order kinetic model demonstrates that liquid-film diffusion acts as the primary rate-limiting step, while the multi-segment linear curves with non-zero intercept from Weber–Morris model confirm that intraparticle diffusion also participates in regulating the overall adsorption rate. Owing to the coexistence of relatively homogeneous Mn–OH active sites introduced by MnO_x_ loading and the intrinsically heterogeneous carbon substrate surface, both Langmuir and Freundlich isotherm equations exhibit high correlation coefficients. The monolayer-adsorption feature originates from limited accessible chemical active sites on the composite surface, whereas the non-ideal heterogeneous-surface adsorption behavior derives from carbon-matrix pores, defects and residual carbon-based adsorption sites. The thermodynamic experiment indicates that the adsorption process is exothermic, and the slight decrease in adsorption uptake with rising temperature is attributed to the enhanced desorption tendency of adsorbed Cd(II) species.

### 3.4. Adsorption Recovery Capacity and Practical Application

The regeneration performance of PEC-MnOx-6 was evaluated by successive adsorption–desorption cycles. After Cd(II) adsorption, the adsorbent was eluted with 0.1 mol/L HNO_3_ solution to desorb Cd(II) from the PEC-MnOx adsorbent. After each adsorption experiment, the spent adsorbent was immersed in the HNO_3_ solution under gentle stirring to remove adsorbed Cd(II). Subsequently, the adsorbent was washed with deionized water until neutral pH, dried, and used for the next adsorption cycle. The measured adsorption uptake retention rate remained above 91.4% after six cycles, which confirms the good reusability of the as-prepared adsorbent. Practical applicability of PEC-MnO_x_-6 was further assessed using authentic water matrices including tap water, river water and sanitary wastewater. High Cd(II) removal efficiencies were achieved across all real-water samples. This observation suggests that the adsorption performance of PEC-MnO_x_-6 would not be compromised by complicated water-matrix components. Accordingly, the as-fabricated composite derived from *Ulva prolifera* displays promising potential for sewage remediation. It not only enables high-value resource utilization of waste algae but also provides cost-effective options for heavy-metal-polluted wastewater treatment.

## 4. Conclusions

Manganese-oxide-modified *Ulva prolifera*-derived porous carbon PEC-MnO_x_ was successfully synthesized via KMnO_4_-assisted pyrolysis activation. The incorporation of manganese oxides not only optimized the pore structure and increased the specific surface area of the carbon matrix but also constructed abundant unsaturated –Mn–OH sites on the material surface, which served as reactive centers for Cd(II) sequestration. Among all prepared samples, PEC-MnO_x_-6 exhibited the best Cd(II) adsorption performance under the optimal conditions of pH = 4 and adsorbent dosage of 0.8 g/L. Kinetic and intraparticle diffusion results demonstrated that Cd(II) uptake was a multi-step mass-transfer process. Liquid-film diffusion acted as the predominant rate-limiting factor, while intraparticle diffusion exerted a synergistic influence on the overall adsorption rate. The comparable fitting performance of Langmuir and Freundlich isotherm equations originated from two co-existing surface features: relatively homogeneous chemical active sites derived from MnO_x_, together with intrinsic heterogeneous pores and structural defects of carbon skeleton. Combined with characterization evidence, Cd(II) immobilization was primarily governed by chemical pathways including surface complexation and ion-exchange. Electrostatic attraction only assisted the enrichment of heavy-metal cations, and physical adsorption induced by van der Waals forces of carbon matrix contributed negligibly. Thermodynamic tests verified the exothermic nature of this adsorption process, where elevated temperature led to a slight decline in adsorption uptake. This work confirms that *Ulva prolifera* biomass can be converted into an efficient heavy-metal adsorbent through facile activation modification, offering a feasible technical reference for *Ulva prolifera* resource utilization and Cd(II)-contaminated wastewater remediation. Further research will be devoted to systematically exploring the potential of PEC-MnO_x_-6 for in situ remediation of Cd(II)-contaminated soil.

## Figures and Tables

**Table 1:** Porous structure parameters of the materials.

Sample	S_BET_ (m^2^/g)	V_tot_ (cm^3^/g)	D_p_ (nm)
PEC	71	2.124	16.02
PEC-MnO_x_-2	585	2.475	27.36
PEC-MnO_x_-4	746	2.525	10.95
PEC-MnO_x_-6	860	2.583	10.36
PEC-MnO_x_-8	806	2.518	8.19

S_BET_: BET surface area; V_tot_: total pore volume; D_p_: average pore size.

## Data Availability

The original contributions presented in this study are included in the article/Supplementary Materials. Further inquiries can be directed to the corresponding author.

## Supplementary Materials

The following supporting information can be downloaded at: https://www.mdpi.com/article/10.3390/molecules31193509/s1, Figure S1. Intraparticle diffusion models for Cd(II) adsorption by (A) PEC, (B) PEC-MnO_x_-2, (C) PEC-MnO_x_-4, (D) PEC-MnO_x_-6, and (E) PEC-MnO_x_-8, respectively. Table S1. The proportion of each element’s atoms in the materials. Figure S2. Cd(II) adsorption capacity of PEC-MnOx-6 for 6 times.
