# Supplementary material for: Manganese-Oxide-Modified Porous Carbon from Ulva prolifera for Efficient Cd(II) Removal: Adsorption Behavior and Mechanisms
Source: Molecules. 2026 Oct 1;31(19):3509. doi: 10.3390/molecules31193509 (PMC13666823; doi:10.3390/molecules31193509)
Supplement: Supplementary file 1 [file molecules-31-03509-s001.zip › molecules-4557753-supplementary.pdf]

Supplementary Materials

Manganese-Oxide-Modified Porous Carbon from *Ulva prolifera* for Efficient Cd(II) Removal: Adsorption Behavior and Mechanisms

Yuhang Zhang, Baoli Wang and Xiujiu Yang *

Haikou Key Laboratory of Marine Contaminants Monitoring Innovation and Application, Haikou Marine Geological Survey, China Geological Survey, Haikou 571127, China; baoli_0916@163.com (B.W.)

* Correspondence: yangxiuj9@163.com

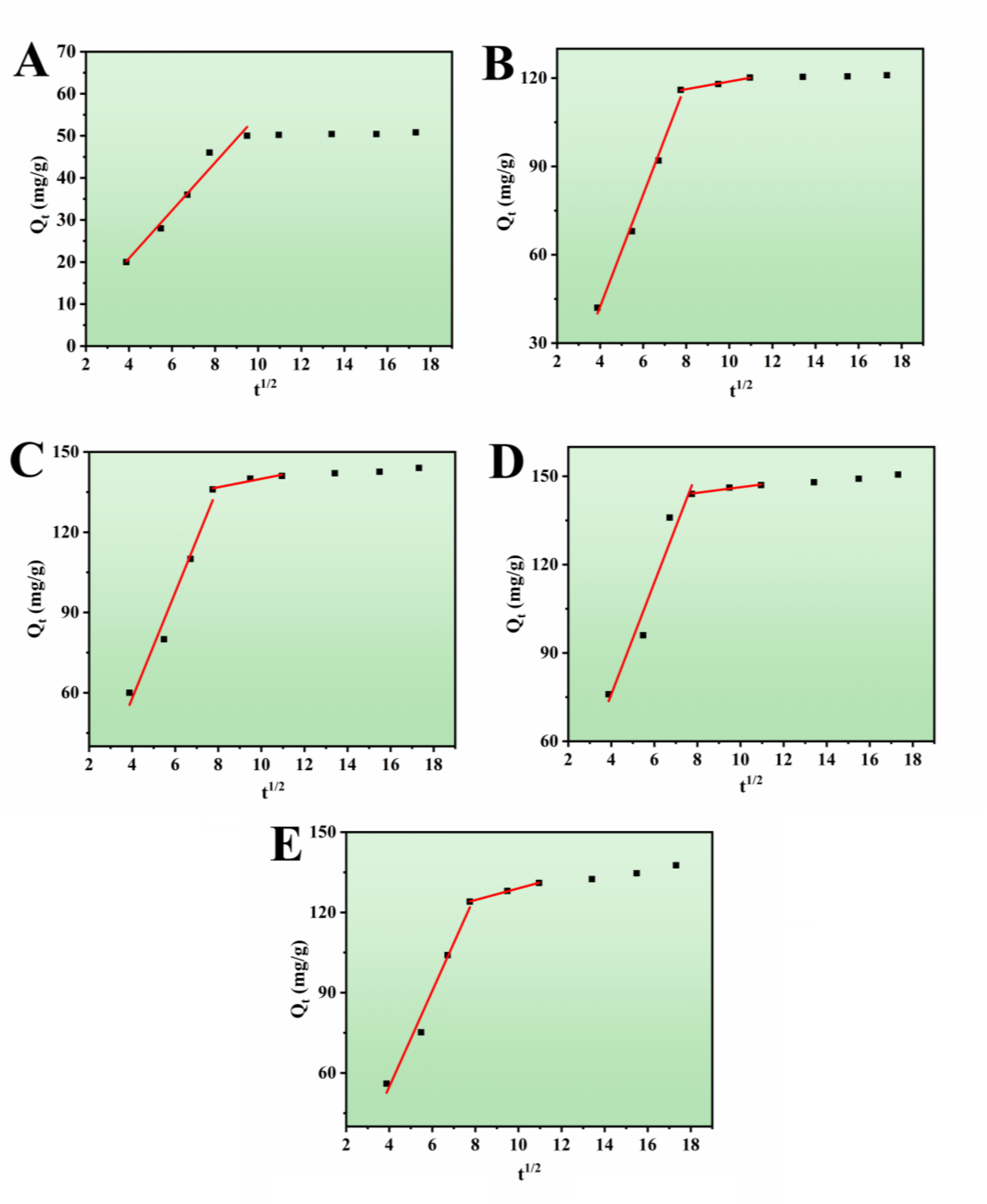

Figure S1. Intraparticle diffusion models for Cd(II) adsorption by (A) PEC, (B) PEC-MnO_x-2, (C) PEC-MnO_x-4, (D) PEC-MnO_x-6, and (E) PEC-MnO_x-8, respectively.

Table S1. The proportion of each element's atoms in the materials.

Sample	C (at%)	Mn (at%)	N (at%)	O (at%)
PEC	87.31	0	2.41	10.28
PEC-MnO_x-2	66.46	2.91	0.11	30.52
PEC-MnO_x-4	64.11	4.52	0.16	31.21
PEC-MnO_x-6	61.88	5.76	0.13	32.23
PEC-MnO_x-8	58.91	6.93	0.09	34.07

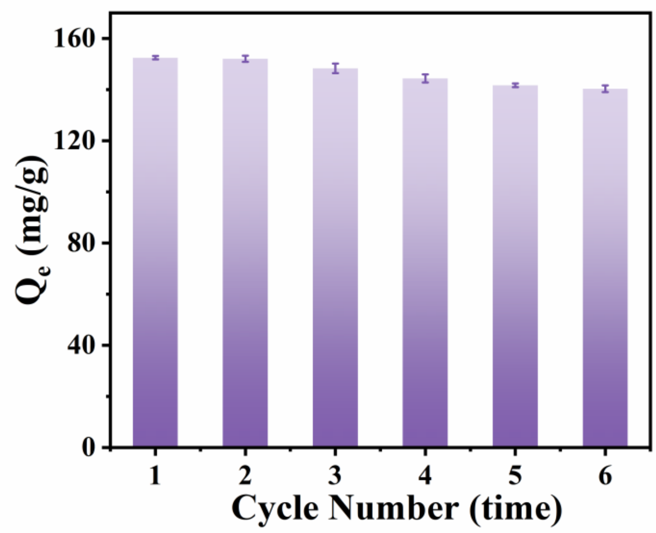

Figure S2. Cd(II) adsorption capacity of PEC-MnO_x-6 for 6 times.
